# Supplementary material for: Cell-Type Specific Neuromodulation of Excitatory and Inhibitory Neurons via Muscarinic Acetylcholine Receptors in Layer 4 of Rat Barrel Cortex
Source: Front Neural Circuits. 2022 Feb 18;16:843025. doi: 10.3389/fncir.2022.843025 (PMC8894850; doi:10.3389/fncir.2022.843025)
Supplement: Supplementary file 1 [file Data_Sheet_1.pdf]

Supplementary figures & tables

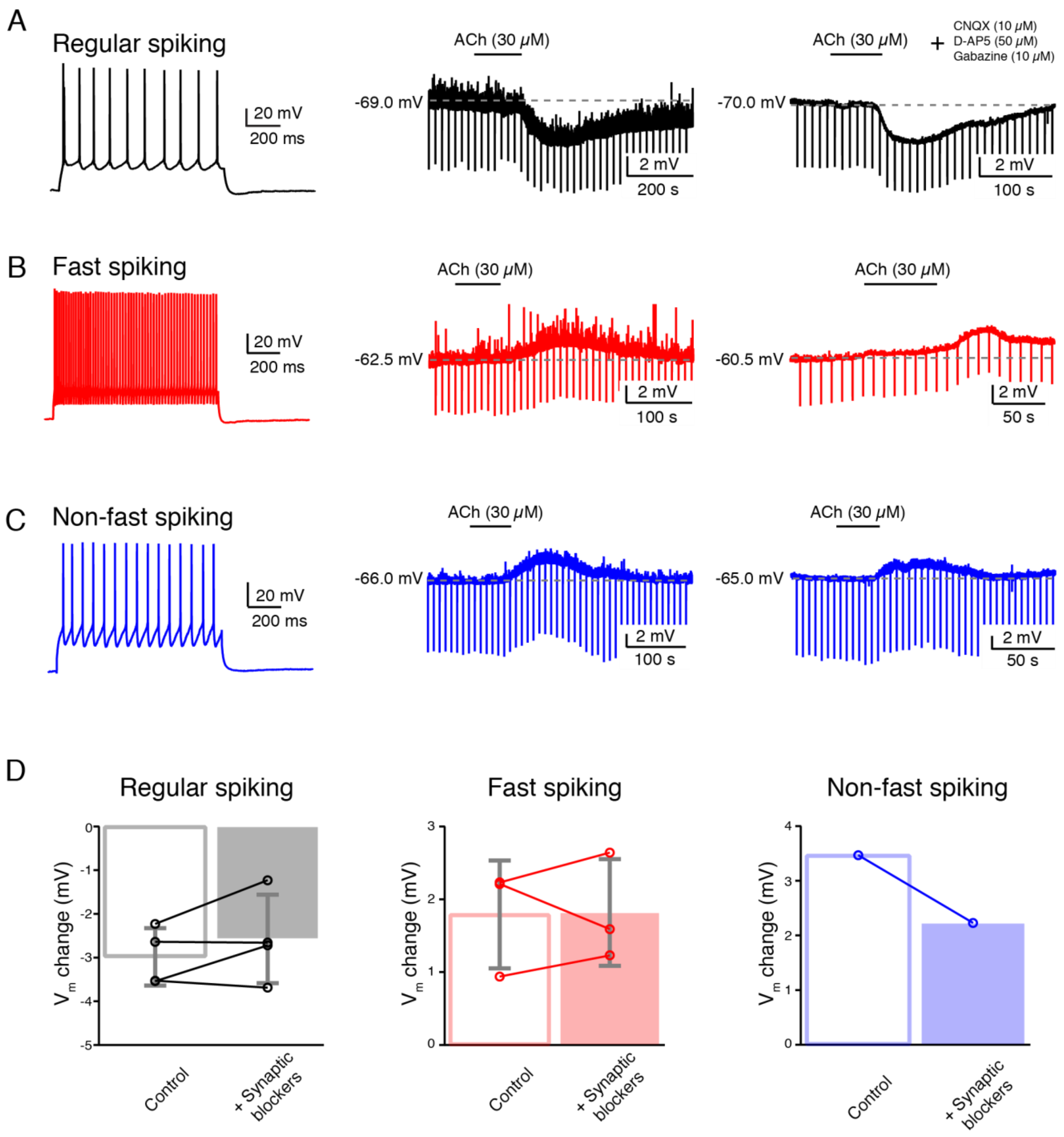

Figure S1. ACh-induced changes in V_m are independent of synaptic transmission.

(A) An example recording of the time course of an ACh-induced Vm change under control condition (middle) and in the presence of a cocktail of synaptic blockers in a L4 RS neuron.

(B) Same as (A) but for a L4 FS interneuron.

(C) Same as (A,B) but for a L4 nFS interneuron.

(D) Comparison histograms of ACh-induced V_m changes for L4 RS (left), FS (middle) and nFS (right) neurons. No statistically significant difference was found.

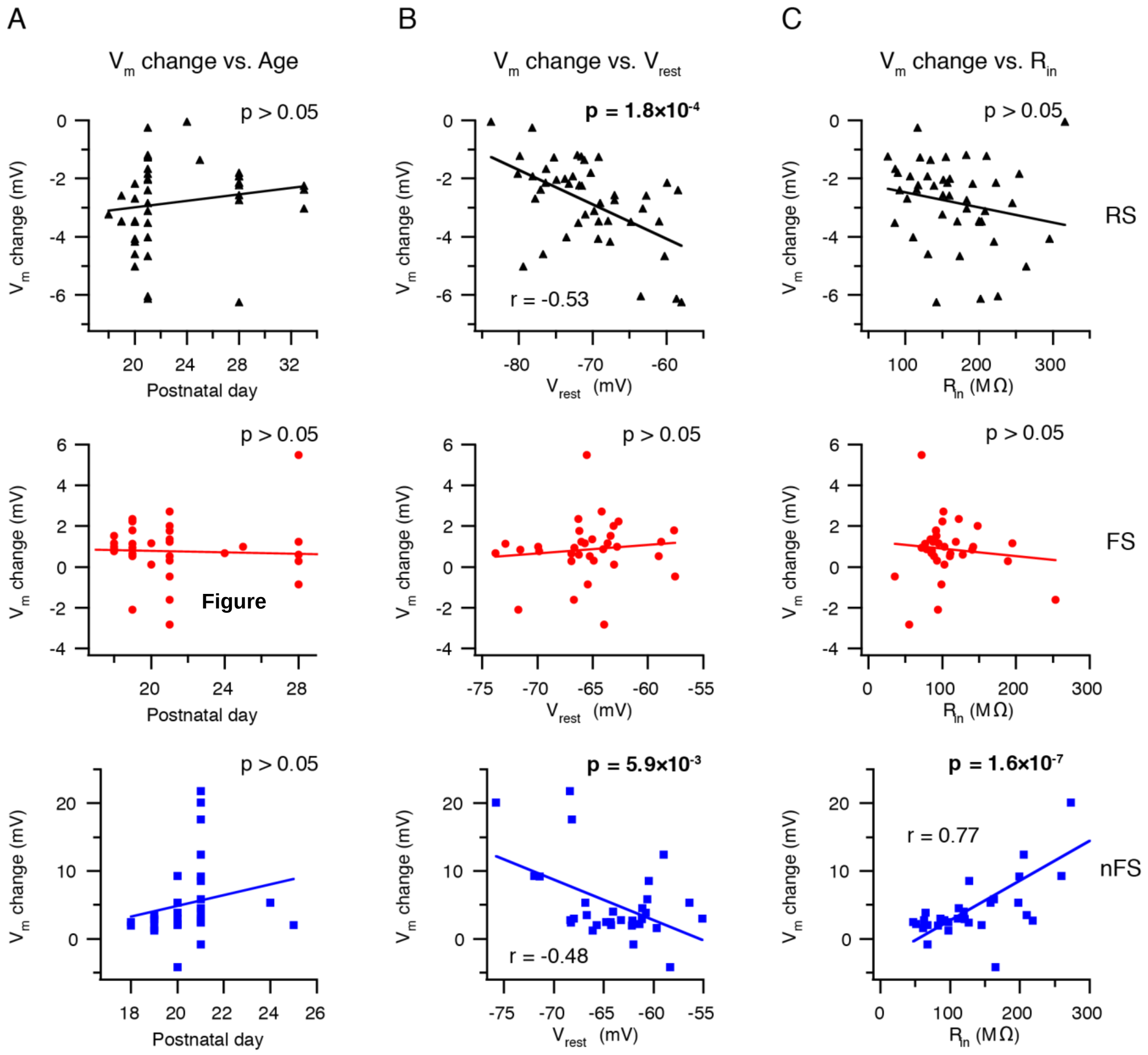

S2. Correlation analysis between the V_m change and the age, the resting membrane potential (V_{rest}) and the input resistance (R_{in}).

(A) Correlation analysis between the V_m change and the age for L4 RS (top), FS (middle) and nFS (bottom) neurons. No statistically significant correlation was found.

(B) Same as (A) but between the V_m change and V_{rest}. Statistically significant correlations were found in L4 RS and nFS neurons.

(C) Same as (A,B) but between the V_m change and R_{in}. A statistically significant correlation was found in L4 nFS neurons.

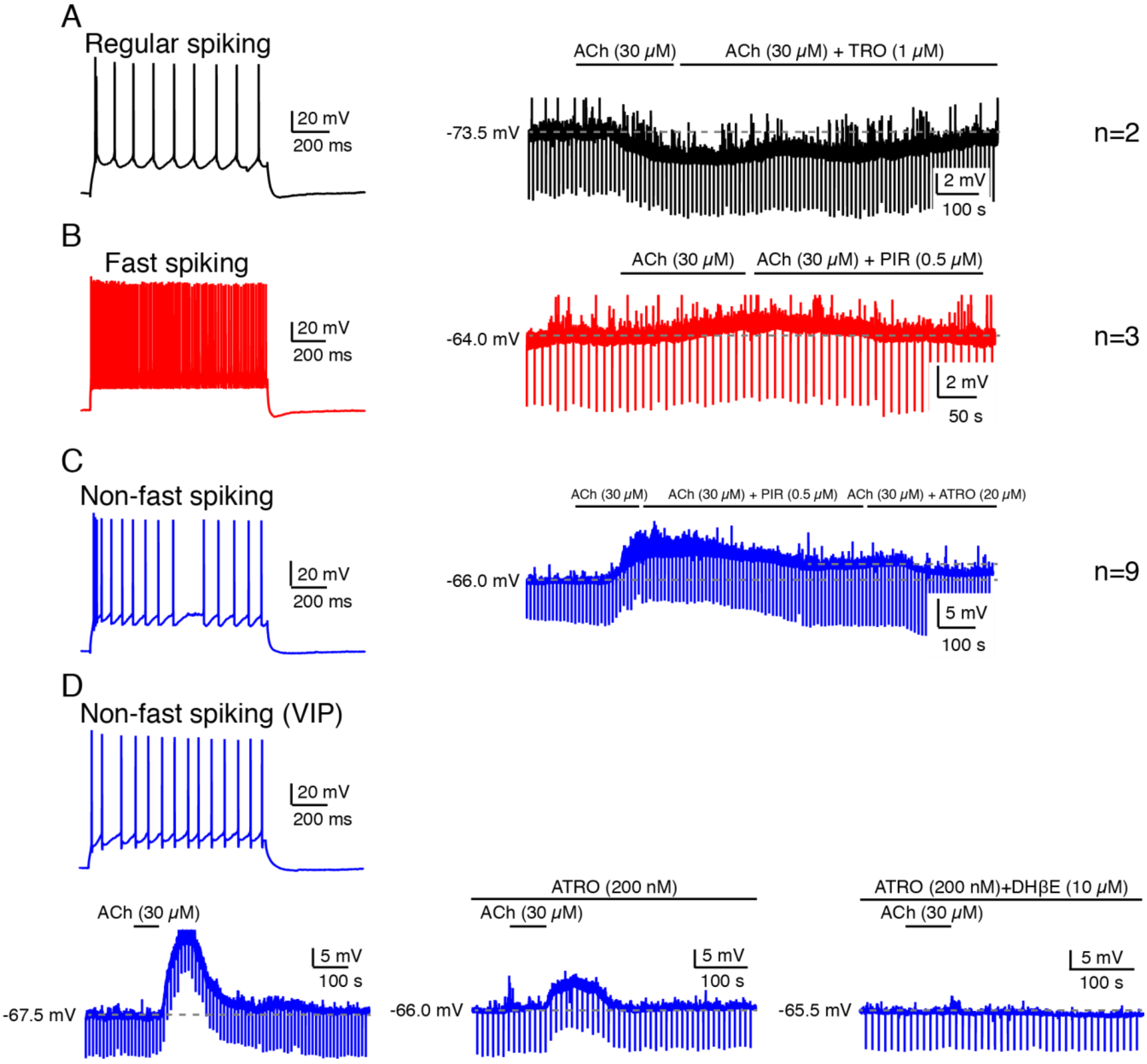

Figure S3.

Example recordings from L4 RS, FS and nFS neurons to identify the subtype of muscarinic and nicotinic receptors that participate in the neuromodulation.

(A) Example recording of the time course of V_m change during the ACh application and during the co-application of ACh and tropicamide, a specific M4 muscarinic receptor antagonist, in a L4 RS neuron. The number of recorded neurons is given on the right.

(B) Example recording of the time course of V_m change during the ACh application and during the co-application of ACh and pirenzepine, a specific M1 muscarinic receptor antagonist, in a L4 FS neuron.

(C) Example recording of the time course of V_m change during the ACh application, during the co-application of ACh and pirenzepine and during the co-application of ACh and atropine in a L4 nFS neuron. Note the residual Vm depolarization during the co-application of ACh and pirenzepine.

(D) Example recording of the time course of V_m change during the ACh application (lower left), during the co-application of ACh and atropine (lower middle) and during the co-application of ACh, atropine and dihydro-ß-erythroidine (lower right), a specific α4β2 subunit-containing nicotinic receptor antagonist, in a L4 nFS neuron.

Table S1. ACh-induced changes in other intrinsic properties of L4 FS and nFS interneurons.

P value was calculated using the non-parametric Wilcoxon signed rank test.

	**L4 FS Ctrl** (n=8)	**L4 FS ACh** (n=8)	**L4 nFS Ctrl** (n=10)	**L4 nFS ACh** (n=10)	**p value (L4 FS Ctrl vs. ACh)**	**p value (L4 nFS Ctrl vs. ACh)**
Passive						
Rin (MΩ)	124.7 ± 64.3	123.8 ± 70.5	123.2 ± 71.2	136.8 ± 43.5	1.00	0.11
Tau (ms)	8.2 ± 1.5	9.3 ± 1.6	11.7 ± 4.9	12.3 ± 4.9	0.44	0.084
Sag (%)	7.6 ± 2.9	3.6 ± 6.5	11.5 ± 6.1	8.0 ± 7.9	0.44	0.28
Single AP						
Rheobase current (pA)	258.3 ± 141.5	225.0 ± 113.8	158.0 ± 75.5	84.0 ± 113.1	0.41	$\mathbf{5.9\times10^{-3}}$
AP threshold (mV)	-31.6 ± 7.0	-32.6 ± 7.0	-36.4 ± 7.1	-35.8 ± 5.7	0.74	0.49
AP half-width (ms)	0.26 ± 0.06	0.26 ± 0.05	0.44 ± 0.10	0.48 ± 0.11	0.31	**0.037**
AP amplitude (mV)	88.0 ± 12.6	80.0 ± 8.9	92.8 ± 10.9	82.6 ± 11.4	0.078	$\mathbf{2.0\times10^{-3}}$
AHP amplitude (mV)	20.4 ± 2.6	20.6 ± 3.9	13.1 ± 4.5	12.7 ± 4.1	0.74	0.77
Repetitive firing						
Max. firing frequency (Hz)	108.5 ± 49.5	126.9 ± 33.2	36.9 ± 11.8	39.8 ± 16.4	0.14	0.98
Slope of F-I curve (APs/100 pA)	37.2 ± 10.3	37.2 ± 8.6	16.1 ± 7.4	21.5 ± 18.1	0.74	1.00
